# PW02-001 - Exome sequencing for autoinflammatory disorders

**DOI:** 10.1186/1546-0096-11-S1-A141

**Published:** 2013-11-08

**Authors:** SE Boyden, Q Zhou, I Aksentijevich, DL Kastner

**Affiliations:** 1 Inflammatory Disease Section, National Human Genome Research Institute, NIH, Bethesda, Maryland, USA

## Introduction

Exome sequencing is the process by which exonic portions of the genome are selectively enriched from genomic DNA samples and sequenced using next generation methodologies. It has been used extensively since 2009 to identify the pathogenic variants underlying Mendelian disorders.

## Objectives

The Inflammatory Disease Section in NHGRI has conducted exome sequencing on 162 subjects from families with a variety of unexplained autoinflammatory, autoimmune, and allergic diseases, to determine the disease etiologies, inform treatments plans, or provide a molecular diagnosis to patients.

## Methods

Samples were prepared at the NIH Intramural Sequencing Center using one of four different exome capture kits, and libraries were sequenced on the Illumina HiSeq 2000 platform using 2x100 bp paired-end reads, to an average depth of coverage in the target intervals of 68X across all samples, and with an average of 89% of target bases producing high-confidence calls. The raw data are analyzed according to the following pipeline: per-sample alignment of reads to the human reference genome with Novoalign and removal of PCR duplicate reads with Picard, followed by multi-sample re-alignment around small insertions and deletions, re-calibration of per-base quality scores, variant calling, and re-calibration of variant quality scores using the Genome Analysis Tool Kit (GATK), and finally variant annotation with Annovar. These steps are performed using the high-performance Biowulf Linux compute cluster at NIH. Generally, annotated variants are filtered to include only those that are nonsynonymous or in splice sites, within linkage intervals (if available), absent from dbSNP v132, have less than 0.1% frequency in 1094 genomes from the 1000 Genomes Project, 6503 exomes from the Exome Sequencing Project, and 938 exomes from the NHGRI ClinSeq project, and co-segregate with the phenotype among all sequenced family members. Putative candidates are then individually examined in the Integrated Genome Viewer (IGV) to eliminate probable false-positives arising from low coverage or mis-aligned reads, and variants passing this check are validated by Sanger sequencing and tested for co-segregation in all available family members.

## Results

We have compared two popular alignment programs, BWA and Novoalign, as well as two popular variant calling tools, SamTools and GATK, and determined that the combination of Novoalign and GATK usually provides the best compromise between specificity and sensitivity for the purposes of Mendelian disease gene identification. Our lab has identified or is currently pursuing the genetic causes of several disorders using the above methodology.

## Conclusion

This approach has been most successful for recessive families with consanguinity or multiple affected individuals, dominant families large enough to produce at least suggestive LOD scores in linkage scans, or families with transmitted *de novo* mutations. For small families and single cases the major challenge is that filtered variant lists contain tens or hundreds of candidates, in which case additional family members or new families with the same phenotype must be collected in order to implicate a single candidate gene.

## Disclosure of interest

None declared.

